# Capsid virus-like particle display improves recombinant influenza neuraminidase antigen stability and immunogenicity in mice

**DOI:** 10.1016/j.isci.2024.110038

**Published:** 2024-05-20

**Authors:** Hyeog Kang, Mira Rakic Martinez, Kara-Lee Aves, Anna Kathrine Okholm, Hongquan Wan, Sylvie Chabot, Tahir Malik, Adam F. Sander, Robert Daniels

**Affiliations:** 1 Division of Viral Products, Center for Biologics Evaluation and Research, Food and Drug Administration, Silver Spring, MD 20993, USA; 2 Department of Immunology and Microbiology, University of Copenhagen, 2200 Copenhagen, Denmark; 3 AdaptVac, Ole Maaløes Vej 3, 2200 Copenhagen, Denmark

**Keywords:** Biological sciences, Immune response, Immunology, Natural sciences

## Abstract

Supplementing influenza vaccines with additional protective antigens such as neuraminidase (NA) is a promising strategy for increasing the breadth of the immune response. Here, we improved the immunogenicity and stability of secreted recombinant NA (rNA) tetramers by covalently conjugating them onto the surface of AP205 capsid virus-like particles (cVLPs) using a Tag/Catcher ligation system. cVLP display increased the induction of IgG2a subclass anti-NA antibodies, which exhibited cross-reactivity with an antigenically distant homologous NA. It also reduced the single dose rNA amounts needed for protection against viral challenge in mice, demonstrating a dose-sparing effect. Moreover, effective cVLP-display was achieved across different NA subtypes, even when the conjugation was performed shortly before administration. Notably, the rNA-cVLP immunogenicity was retained upon mixing or co-administering with commercial vaccines. These results highlight the potential of this approach for bolstering the protective immune responses elicited by influenza vaccines.

## Highlights

• cVLP-display increases rNA immunogenicity and reduces the protective dose
• Coupling to cVLPs stabilizes and maintains rNA potency for at least 10 months at 4°C
• cVLP-displayed rNA can be co-administered with commercial HA-based influenza vaccines

## Graphical abstract

**Figure:**
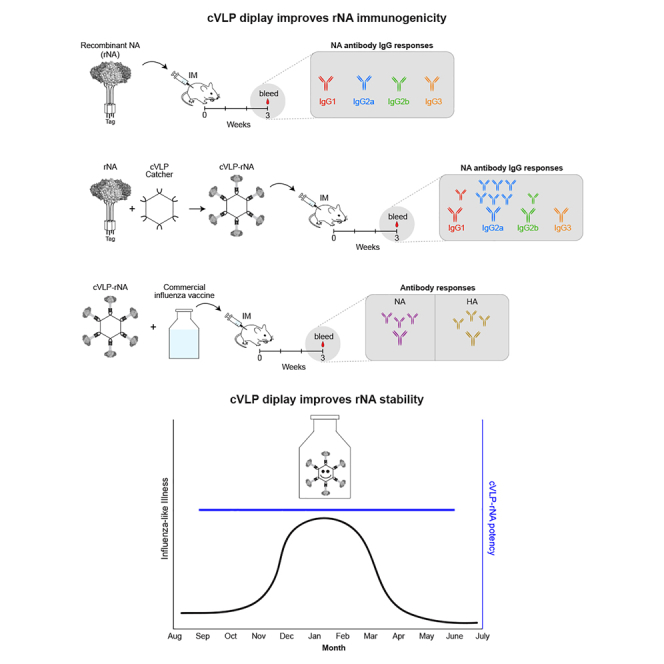

## Introduction

Although multiple vaccines are available, influenza viruses continue to cause significant health and economic impacts.^1^ This is partly because current influenza vaccines fail to elicit broad and durable protective immunity,^2^^,^^3^ resulting in suboptimal efficacy that varies between seasons and rarely exceeds 60% in the U.S..^4^ Despite these shortcomings, influenza vaccines have undergone few changes and remain focused on optimizing neutralizing antibody responses against the major surface antigen, hemagglutinin (HA).^5^^,^^6^ However, the consistently low efficacy of influenza vaccines has started to challenge this approach, leading to a growing interest in combining HA with other influenza antigens such as neuraminidase (NA) to create new and more efficacious vaccines.

NA is a labile tetrameric enzyme^7^ that promotes viral movement through the respiratory tract mucosa by removing the terminal sialic acid receptors for the more abundant HA.^8^^,^^9^^,^^10^^,^^11^ The concept of supplementing influenza vaccines with additional NA antigen is largely based on its low abundance in viruses, which are commonly used as vaccine antigen sources, combined with work demonstrating NA antibody responses are protective in humans^12^^,^^13^ and multiple animal models.^14^^,^^15^^,^^16^^,^^17^^,^^18^ More recent mechanistic studies have shown that anti-NA antibodies can provide protection by inhibiting NA’s ability to cleave sialic acid on complex glycans or by activating antibody-dependent cellular cytotoxicity and phagocytosis via the Fcγ receptor.^19^^,^^20^^,^^21^^,^^22^^,^^23^^,^^24^^,^^25^ Rather than using full-length recombinant NA (rNA) in detergent micelles, similar to recombinant HA antigens in FluBlok,^26^ most of the recent studies on NA immune responses were performed with secreted rNA chimeras that use a stabilizing tetramerization domain as a functional surrogate for the transmembrane domain.^17^^,^^18^^,^^27^^,^^28^^,^^29^ However, vaccination schemes with these rNAs often require multiple immunizations with high doses and the addition of adjuvant, suggesting their immunogenicity in naive animals is low.

Soluble antigens such as secreted rNA chimeras often are unable to effectively engage the immune system in a manner that elicits robust humoral responses.^30^ Consequently, several approaches have been developed to increase the immunogenicity of soluble recombinant antigens by mimicking the multivalent antigen presentation of a virus. These include conjugating antigens on the surface of nanoparticles such as capsid-based virus-like particles (cVLPs),^31^ and binding antigens to the surface of liposomes.^32^ cVLPs are non-infectious protein nanostructures that possess several key characteristics, making them effective vaccine scaffolds. These include their larger size (≥10 nm) and particulate nature, *in vivo* kinetics, and highly repetitive surface geometry. Together, these factors stimulate the innate immune system, increase drainage to the lymph nodes and enhance B cell receptor cross linking, resulting in stronger activation.^33^

Recent studies have shown that recombinant vaccine antigens displayed on the surface of cVLPs can inherit these immunologically favorable properties and elicit strong antibody responses.^34^^,^^35^^,^^36^^,^^37^^,^^38^ In general, recombinant antigens have been added to cVLPs either by using genetic fusions or modular approaches where antigens are added to preassembled cVLPs by chemical crosslinking or tag coupling.^38^ While genetic fusions can result in high-density antigen presentation, they are dependent on the ability of the fusion protein to retain the native antigen structure and assemble the cVLP, which can be challenging for complex homo-oligomeric antigens such as rNA. To overcome this issue, previous studies have used chemical crosslinking to create protein nanoparticle vaccines that display rNAs on the surface.^39^^,^^40^ However, the nonspecific nature of chemical crosslinking introduces inherent heterogeneity, making it challenging to achieve the consistency needed for a vaccine.

Bioconjugation approaches like the Tag/Catcher AP205 cVLP system can covalently couple large structurally complex antigens onto cVLPs in a controlled and directional manner.^31^^,^^35^^,^^41^^,^^42^ Similar to SpyCatcher-SpyTag,^43^ this system relies on a split-protein conjugation where the antigen is expressed with a short peptide Tag that rapidly forms an irreversible isopeptide bond upon association with a Catcher binding partner. On AP205, Catchers are evenly distributed on the surface to present conjugated antigens in a uniform orientation that mimics the multivalent display on a virus. In addition, recent Phase I-III human clinical trials have shown that this display platform is safe and capable of eliciting robust and durable neutralizing antibody responses against a recombinant SARS-CoV-2 antigen,^44^ suggesting it could be used to develop an effective rNA vaccine with improved immunogenicity.

Recently, we observed that inactivated virions elicit strong antibody responses despite having low NA content,^45^ suggesting the immunogenicity of soluble rNAs could be enhanced by mimicking the multivalent display of NA on a virus. Here, we examined the immunogenicity of soluble rNA tetramers that were covalently coupled onto the surface of AP205 cVLPs by a Tag/Catcher bioconjugation system. The multivalent display on the cVLPs significantly improved the rNA stability and immunogenicity, resulting in a dose sparing effect in a mouse viral challenge model. The cVLP display approach biased the production of IgG2a antibodies against NA that showed cross-reactivity with an antigenically distant homologous NA and was adaptable to rNAs from subtype 1 (rN1) and 2 (rN2). In addition, the enhanced immunogenicity was retained following different supplementation strategies with commercial influenza vaccines, illustrating how this approach can be utilized to potentially increase the breadth and efficacy of the immune response to influenza vaccines.

## Results

### Comparison of antibody responses against soluble recombinant neuraminidase and full-length neuraminidase in virions

**Figure 1:**
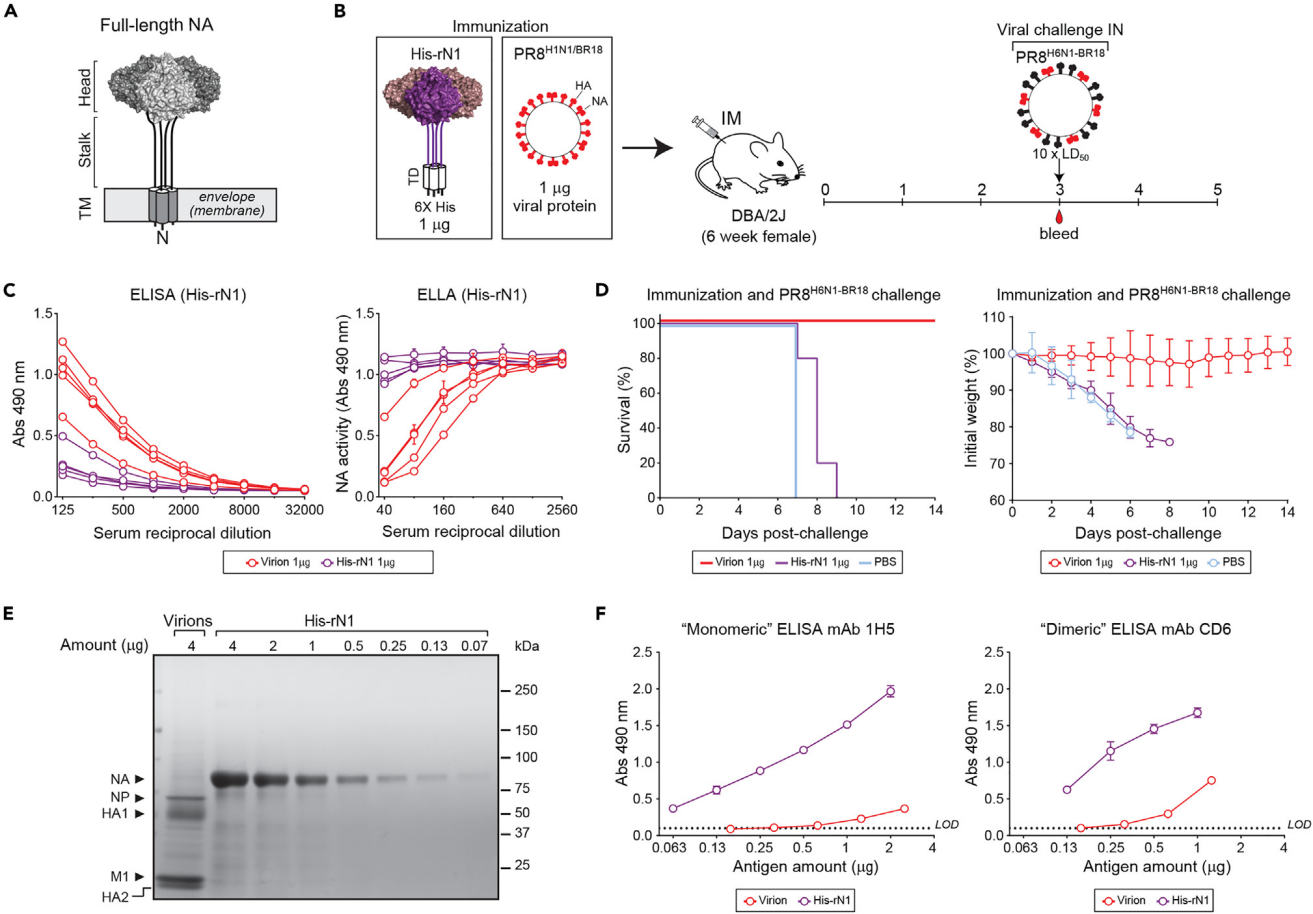
Immunization with inactivated virions versus recombinant NA protein in mice (A)Diagram of a full-length NA tetramer showing the enzymatic head, stalk, and N-terminal transmembrane (TM) domains. (B) Scheme for evaluating NA antibody responses in groups of female DBA/2J mice (*n* = 5) after intramuscular (IM) immunizations with 1 μg of a soluble rNA (His-rN1) protein stabilized by an N-terminal tetramerization domain (TD) or BPL inactivated PR8^H1N1/BR18^ virions carrying an identical NA. Three weeks post-immunization mice were bled and intranasally (IN) challenged with a 10× median lethal dose (LD_50_) of recombinant PR8^H6N1−BR18^ virus. (C) Sera collected 3 weeks post-immunization were assessed for NA antibodies by ELISA (left panel) and NAI antibodies by ELLA using His-rN1 protein (right panel). ELISA and ELLA curves from each sera are shown. ELISA results for each sera are shown and the ELLA results are plotted as the mean of duplicate samples ± standard deviation (SD). (D) Graphs displaying the survival (left panel) and mean weight loss ±SD (right panel) in each group at the indicated times post-challenge. (E) PR8^H1N1/BR18^ virions (4 μg total protein) were resolved by reducing Coomassie stained SDS-PAGE (12-4% gel) along with the indicated His-rN1 amounts. (F) Relative NA amounts in the PR8^H1N1/BR18^ virions and His-rN1 preparation were analyzed by “Monomeric” (left panel) and “Dimeric” (right panel) N1 capture ELISAs that used the mAbs 1H5 or CD6, respectively. Data are the mean ±SD of n *= 1* biologically independent experiments run in triplicate.

Improving the immunogenicity of rNAs could significantly aid in the development of approaches for supplementing influenza vaccines with NA. In a previous study, we observed that inactivated virions elicit strong antibody responses against NA,^45^ suggesting that the viral display can contribute to the immunogenicity of full-length NA tetramers (Figure 1A). To initially test this possibility, we intramuscularly immunized mice with 1 μg of inactivated H1N1/BR18 virions (PR8^H1N1/BR18^) that were generated with an A/Puerto Rico/8/1934 (PR8) backbone or 1 μg of an identical soluble His-tagged rNA (His-rN1) and challenged the mice with a lethal dose of a virus (PR8^H6N1−BR18^) that encodes the same NA and a different HA (H6) subtype (Figure 1B). Three weeks post-immunization, NA binding antibody titers were measured by ELISA and NA inhibiting (NAI) antibody titers were measured by an enzyme-linked lectin assay (ELLA) using His-rN1 protein (Figure 1C) and virions (Figure S1). In all assays, sera from the inactivated virion immunized group displayed higher NA binding and NAI antibody titers than the His-rN1 and PBS control groups. In line with the serology, only the inactivated virion immunized mice survived the challenge and showed minimal weight loss (Figure 1D).

Densitometry analysis of Coomassie stained SDS-PAGE gel analysis, which used the His-rN1 as a standard, indicated that NA only accounts for ∼3% of the total viral protein in the PR8^H1N1/BR18^ virions (Figure 1E). Similar results were obtained from an ELISA with the monoclonal antibody (mAb) 1H5^46^ which recognizes an epitope in the head domain of an N1 monomer (Figure 1F, left panel). When the ELISA was performed with the mAb CD6,^19^ which recognizes N1 “dimers” or the interface of two N1 monomers (Figure 1F, right panel), NA accounted for 10% of the total viral protein, indicating the fraction of properly assembled NA may be greater in the virions than the His-rN1 preparation. Together, these results suggest that the high immunogenicity of NA in a virion is not quantity related, but rather due to the context of the presentation to the immune system which includes the virion size, multivalent NA display in a fixed topology, and the presence of additional immunostimulatory viral components.

### Production and characterization of capsid virus-like particles displaying rN1 on the surface

**Figure 2:**
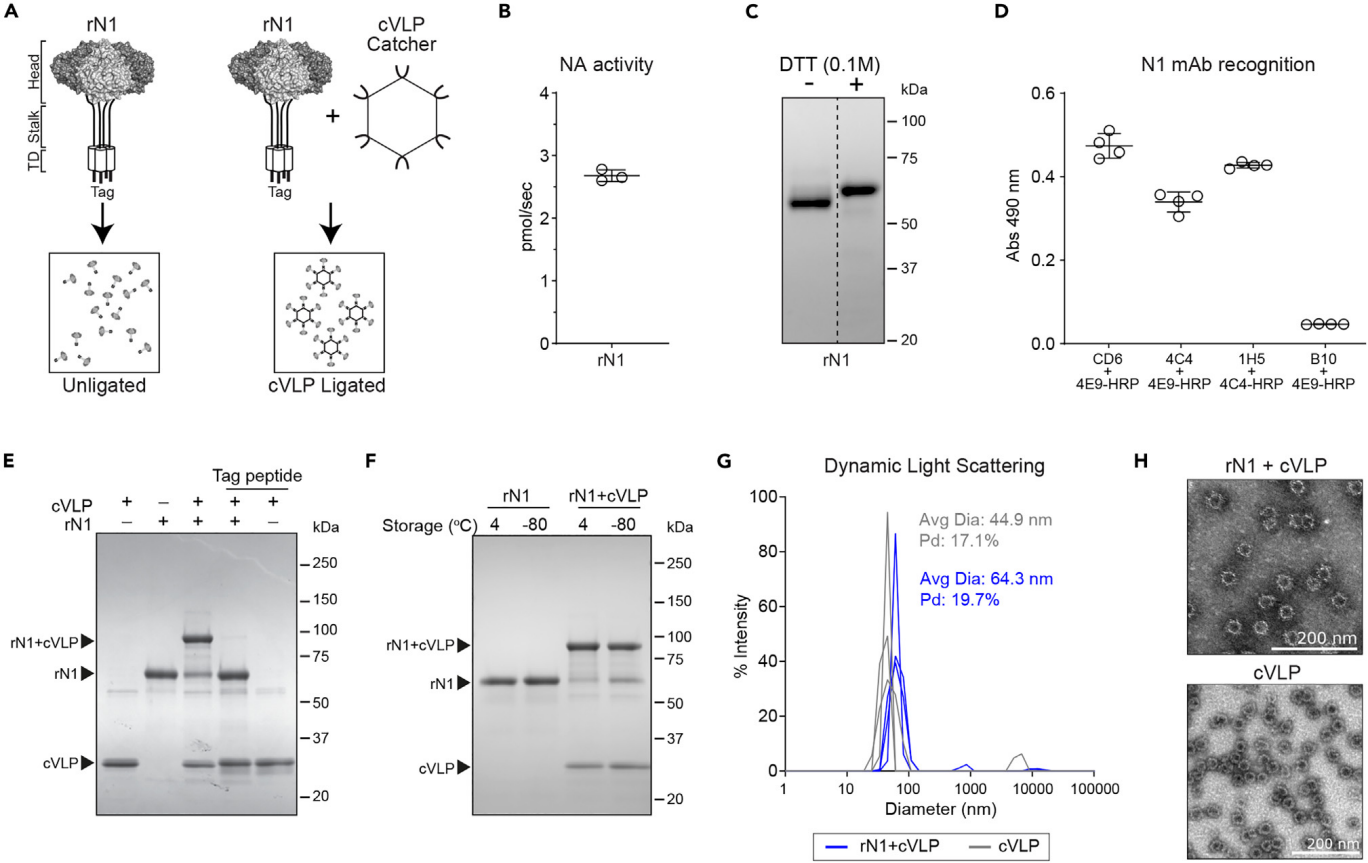
Characterization of rN1 before and after ligation to AP205 cVLP Catchers (A) Schematic of the rNA (rN1) that was designed with an *N*-terminal Tag to facilitate ligation to AP205 cVLPs. (B) Graph displaying the specific activity in ∼50 ng of rN1. Activity measurements from n *= 3* biologically independent experiments are shown with the mean (bar) ± SD. (C) Representative image of rN1 (∼2 μg) resolved by SDS-PAGE with or without DTT and visualized by Coomassie staining. (D) Epitope conservation in the rN1 was examined by sandwich ELISAs using different N1 head domain-specific mAbs (CD6, 4C4, 1H5, and 4E9). The N2-specific mAb B10 is included as a control. Data from n *= 2* biologically independent experiments run in duplicate using 100 ng of rN1 are shown with the mean (bar) ± SD. (E) AP205 cVLPs were incubated with a 1:1 M ratio of Tag-rN1 for 18 h at 4°C. Where indicated a Tag-peptide was added at a 20:1 M ratio with the cVLPs and incubated prior to rN1 addition. Samples (2 μg) were resolved by reducing SDS-PAGE and visualized by Coomassie staining. Bands corresponding to the cVLP, rN1, and cVLP ligated rN1 are indicated. (F) rN1 was incubated at a 1:1 M ratio with AP205 cVLPs for 18 h at 4°C or mock incubated prior to storage at 4°C or −80°C. Four days post-ligation, samples (2 μg) were resolved by reducing SDS-PAGE and visualized by Coomassie staining. (G) Size distributions of rN1 ligated AP205 cVLPs stored at −80°C were measured by dynamic light scattering and compared to unligated AP205 cVLPs. Three independent measurements are displayed with the average diameter and polydispersity (Pd) of the major peak. (H) Representative TEM images of rN1 ligated cVLPs and unligated cVLPs stored at −80°C are shown.

Tag/Catcher cVLPs have previously been shown to mediate the display of large oligomeric antigens in a high-density, unidirectional format,^35^^,^^47^ making it an ideal system to examine if multivalent viral-like display can improve the immunogenicity of soluble rNA antigens (Figure 2A). To implement this system, we used a multistep chromatography procedure^48^ to purify a secreted rN1 chimera with a 15-residue split-protein binding Tag on the N-terminus. The isolated rN1 was enzymatically active (Figure 2B), showed high purity on Coomassie stained SDS-PAGE gels (Figure 2C) and was recognized by several N1 specific mAbs (Figure 2D), indicating the rN1 was a functional tetramer and retained antigenic epitopes. We then incubated increasing rN1 amounts with AP205 cVLPs overnight at 4°C and determined that a molar ratio of 1:1 resulted in the display of up to 45 rN1 tetramers per cVLP, while minimizing the amount of unligated rN1 (Figures S2A–S2C). The cVLP ligation did not affect rN1 activity (Figure S2D) and we observed similar kinetics with a subtype 2 rNA (Figure S3), indicating the rNA structures remained intact and that the NA sequence does not influence the ligation. Furthermore, preincubation of cVLPs with a 15-residue binding Tag peptide inhibited the rN1 ligation (Figure 2E), showing the ligation was specific.

Since Tag/Catcher ligations are time and temperature-dependent (Figure S4), larger scale cVLP ligations with rN1 were performed at 4°C overnight using a 1:1 M ratio and multiple aliquots were stored at 4°C and −80°C. Apart from slightly higher amounts of ligated rN1 at 4°C after 4 days, the protein profiles by SDS-PAGE were similar for the two storage conditions (Figure 2F). Dynamic light scattering (Figure 2G) and transmission electron microscopy (Figure 2H) confirmed that the cVLP size increased following the rN1 ligation and that the structural integrity remained intact, whereas size exclusion chromatography showed that the majority of rN1 coeluted with the cVLPs after ligation (Figure S5).

**Figure 3:**
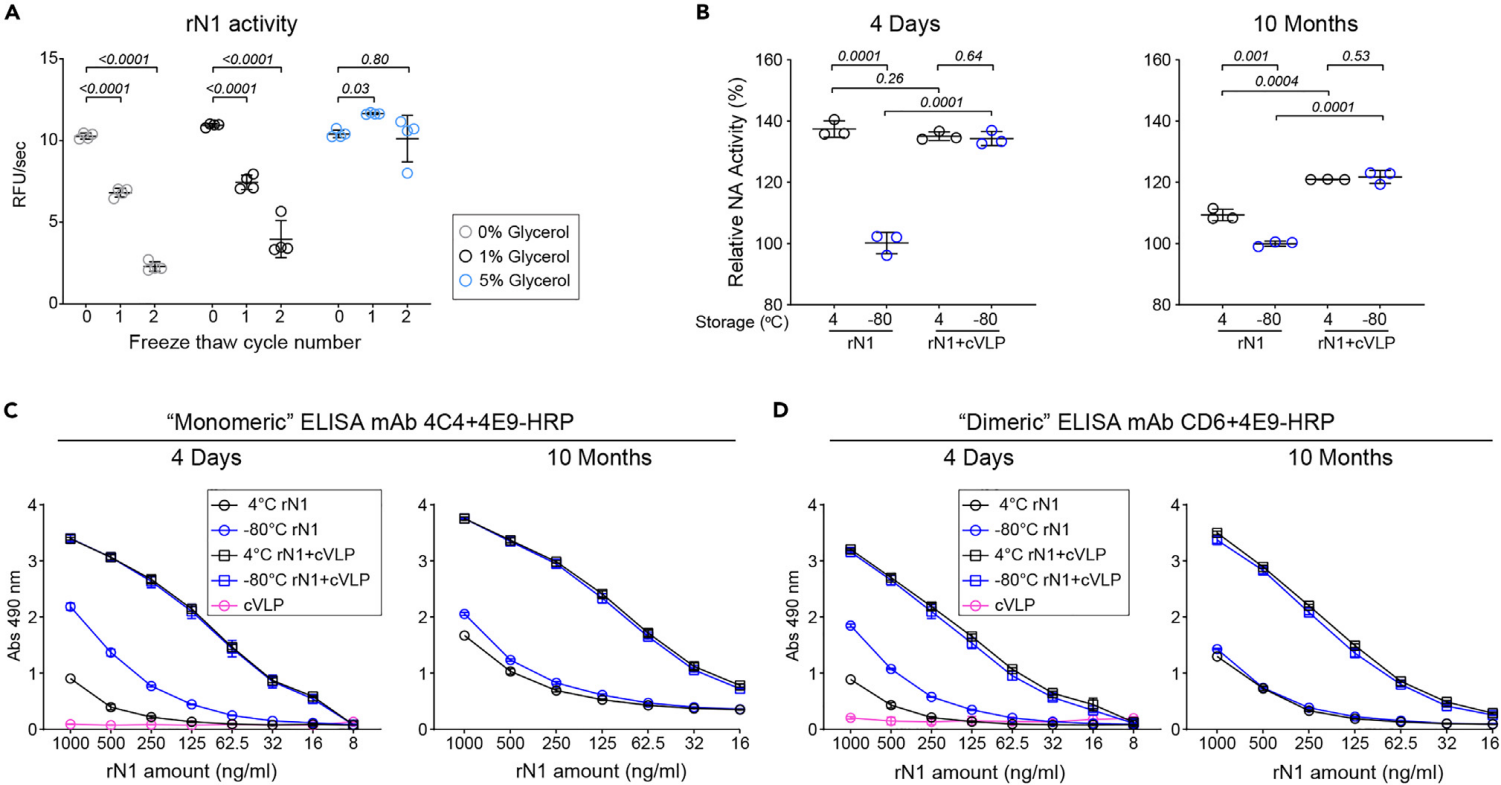
cVLP ligation improves soluble rN1 stability (A) rN1 was adjusted to 0, 1, or 5% glycerol and subjected to the indicated number of freeze thaw cycles prior to measuring NA activity in equal protein amounts (∼50 ng) using MUNANA. Data from n *= 2* biologically independent experiments performed in duplicate are shown with the mean (bar) ± SD. *p* values were determined using a two-way ANOVA Tukey multiple comparisons test with single pooled variance and a 95% confidence interval (CI). (B) NA activities were measured in mock and AP205 cVLP ligated rN1 preparations following storage at 4°C or −80°C for 4 days (left panel) and 10 months (right panel). Data from n *= 1* biologically independent experiments run in triplicate using ∼50 ng of rN1 are shown with the mean ± SD. Mean activity of the mock ligated rN1 stored at −80°C was set to 100% for each time point. *p* values were determined by a student unpaired t-test with a 95% CI. (C and D) “Monomeric” (C) and “Dimeric” (D) N1 capture sandwich ELISAs were used to monitor antigenic changes in rN1 following cVLP ligation and storage at 4°C or −80°C for 4 days and 10 months. Data are displayed as the mean ± SD from n *= 3* biologically independent experiments.

Ideal influenza vaccines should display stable quality attributes for an entire influenza season or longer and rNA is a labile protein that generally shows activity loss following freeze-thawing in the absence of stabilizing agents such as glycerol (Figure 3A). Therefore, we measured the rN1 activity and the epitope integrity of the samples (rN1 alone or cVLP ligated) stored at 4°C and −80°C for 4 days and 10 months to account for the duration of an influenza season. All 4-day samples showed identical activity except for the rN1 stored at −80°C, which was attributed to freeze-thawing (Figure 3B, left panel). After 10 months the activity of the cVLP-ligated rN1 remained identical in both storage conditions while the activity of the rN1 stored at 4°C reduced toward the −80°C stored samples (Figure 3B, right panel), indicating cVLP ligation increases soluble rN1 stability.

Analysis of the 4-day and 10-month samples by N1 “monomeric” (Figure 3C) and “dimeric” (Figure 3D) ELISAs yielded similar results, supporting the conclusion that cVLP ligation increased rN1 stability. At both times, no significant difference was observed in the cVLP-ligated rN1 stored at 4°C and −80°C, indicating the rN1 epitope presentation did not change. In contrast, both 4-day rN1 ELISAs showed stronger signal for the −80°C stored samples, likely due to the freeze thaw induced structural alterations. After 10 months, the signal from the rN1 samples stored at 4°C increased and the difference disappeared, suggesting the structure of the 4°C stored rN1 had changed over time, a conclusion in line with previous data^28^ and supported by a similar observation with a soluble His-tagged version of rN1 (Figure S6). Together, these results demonstrate that cVLP ligation stabilizes the presentation of soluble rN1 epitopes at 4°C and during freeze thawing, which can help to extend the shelf-life of a rNA vaccine by maintaining the rNA antigen consistency over time.

### Multivalent display on capsid virus-like particles improves soluble recombinant neuraminidase immunogenicity

**Figure 4:**
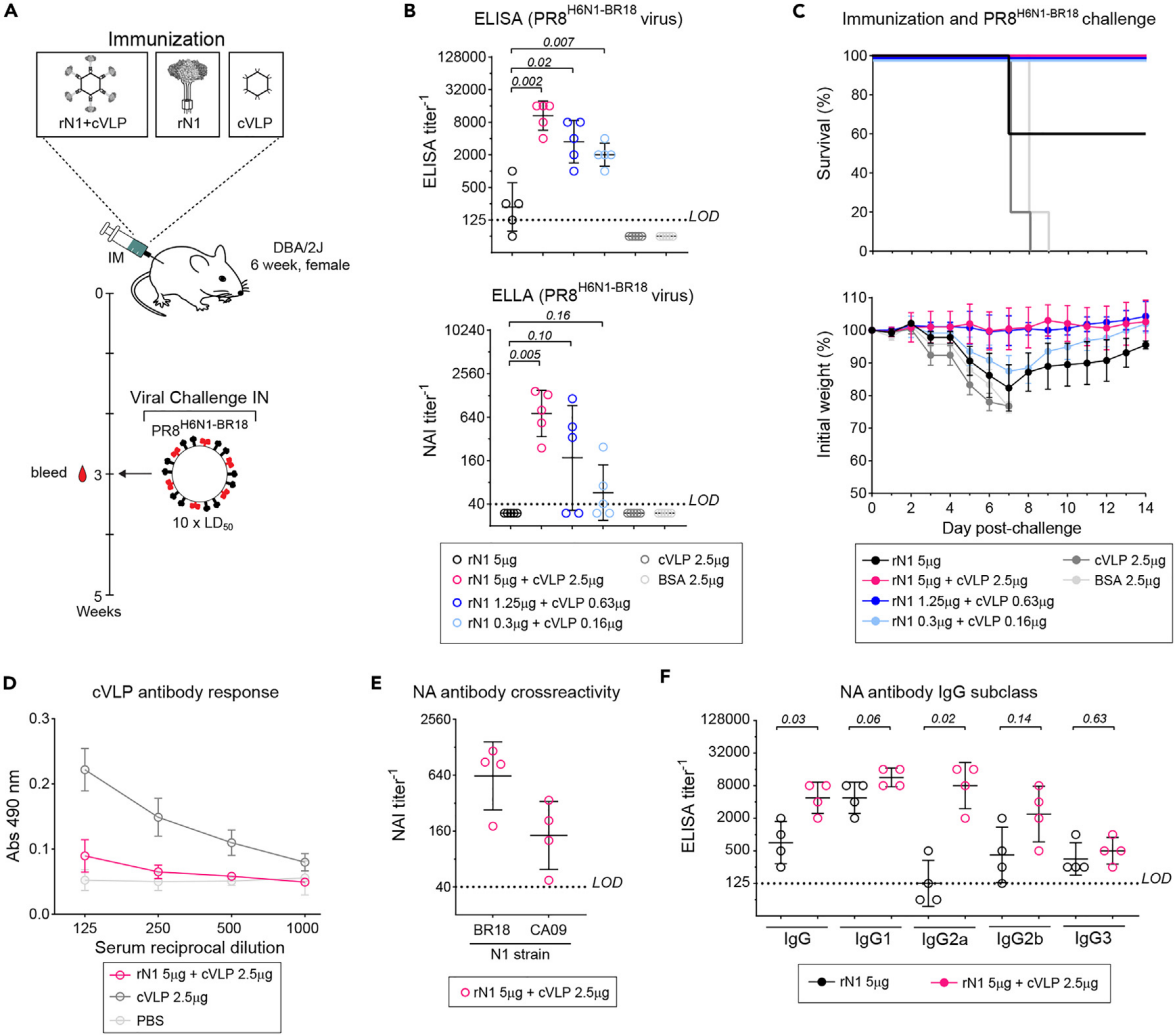
Ligation to AP205 cVLPs increases rN1 immunogenicity in mice (A) Diagram of the scheme for comparing the immune responses to unligated and AP205 cVLP ligated rN1 in groups of female DBA/2J mice (*n* = 5). (B) NA sera antibody titers in each group were assessed 3 weeks post-immunization by an endpoint ELISA (top panel) and NAI antibody titers were measured by ELLA (bottom panel). Titers were determined with recombinant PR8^H1N1/BR18^ virus and are displayed with the geometric mean (bar) ± SD. *p* values were calculated by a student unpaired t-test with a 95% CI by assigning 0 to samples below the limit of detection (LOD). (C) Graphs displaying the survival (top panel) and mean weight loss ±SD in the immunization groups following the PR8^H6N1−BR18^ viral challenge. (D) Antibody responses against the cVLP were measured by a direct ELISA using wells coated with unligated cVLP. Mean values from the indicated groups are displayed ±SD. (E) N1 cross reactivity was examined by measuring sera NAI titers by ELLA using viruses carrying either N1/BR18 or N1/CA09. Individual sera titers are displayed with the geometric mean (bar) ± SD. (F) NA sera antibody titers for the indicated IgG subclasses were measured in groups of mice (*n* = 4) at 3 weeks post-immunization by an endpoint ELISA using PR8^H6N1−BR18^ virus and are displayed with the geometric mean (bar) ± SD. *p* values were calculated by a student unpaired t-test with a 95% CI by assigning 0 to samples below the LOD.

To determine if the large multivalent cVLP display enhances soluble rNA antibody responses and protection, we immunized groups of mice with three different dose amounts of cVLP ligated rN1 and subjected them to a lethal viral (PR8^H6N1−BR18^) challenge (Figure 4A; Table 1). The control groups included BSA and the highest amount of rN1 (5 μg) or cVLPs (2.5 μg). Three weeks post-immunization the cVLP-ligated rN1 groups displayed dose-dependent increases in NA binding (Figure 4B, upper panel) and NAI (Figure 4B, lower panel) antibody titers that were higher or equivalent to the rN1 (5 μg) control group at all three dose amounts. Supporting the serology, all mice that received cVLP-ligated rN1 survived the challenge (Figure 4C, upper panel) and displayed a dose-dependent change in weight loss (Figure 4C, lower panel). In the controls, only 3 mice from the rN1 (5 μg) group survived the challenge, but these mice showed similar to more severe weight loss than the group that received a 16-fold lower dose of rN1 ligated to cVLPs. Similar results (increased NA antibody titers and survival from challenge) were also observed when we compared unligated and cVLP-ligated rN2 (Figure S7), demonstrating the general benefit of the multivalent display for soluble rNA antigens.

**Table 1:** Endotoxin, DNA, RNA and purity measurements in the rNA ligated cVLP doses

	High Dose			Mid Dose			Low Dose		
	cVLP (2.5 μg)	rN1 (5μg)	Total^a^	cVLP (0.63 μg)	rN1 (1.25μg)	Total^a^	cVLP (0.016 μg)	rN1 (0.03μg)	Total^a^
Endotoxin (EU)	0.15	0.29	0.44	0.038	0.073	0.11	0.009	0.018	0.027
DNA (ng)	<10	<10	<20	<2.5	<2.5	<5.0	<0.63	<0.63	<1.25
RNA (ng)	161	<10	<171	40.25	<2.5	<42.8	10.1	<0.63	<10.7
Purity^b^	>95%	>95%	ND	ND	ND	ND	ND	ND	ND

ND, not determined.

a Calculated by combining the measurements of the individual component.

b Determined by Coomassie stained gel densitometry for the individual components.

Compared to the cVLP control group, sera from the high dose rN1 ligated cVLP group showed much lower antibody responses to the cVLPs by ELISA (Figure 4D), indicating that the multiple copies of covalently bound rN1 tetramers likely minimize the exposure of cVLP surface epitopes. The NAI antibodies elicited by the rN1 ligated cVLPs also cross reacted with an earlier antigenically distinct N1 (A/California/04/2009),^49^ yielding slightly lower NAI titers (Figure 4E), demonstrating the increased immunogenicity did not reduce the breadth of the antibody response. Finally, an IgG subclass analysis revealed that the cVLP ligation biased the production of NA-specific IgG2a antibodies (Figure 4F), likely due to the TLR7 stimulatory effects of the prokaryotic RNA encapsulated inside the cVLPs (Table 1; Gomes et al.^50^).

### Capsid virus-like particle display of rN1 supports multiple admix strategies that include commercial vaccines

**Figure 5:**
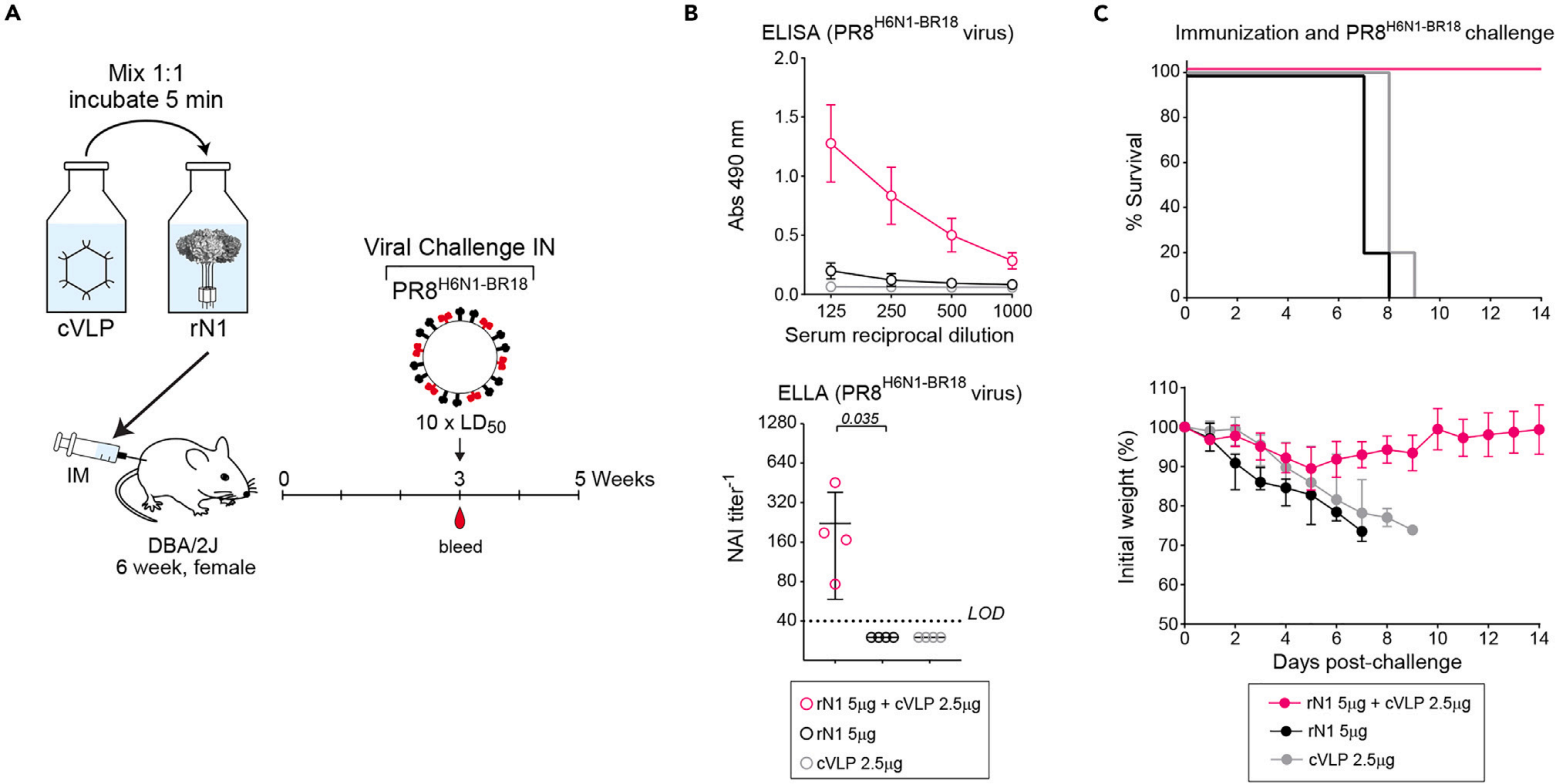
Immunogenicity of rN1 ligated with cVLPs in an admix approach (A) Diagram of the admix immunization scheme using rN1 and AP205 cVLPs in groups of female DBA/2J mice (*n* = 4). Vials containing rN1 (0.2 mg/mL) and cVLPs (0.1 mg/mL) were mixed 1:1 (molar ratio of ∼1) and incubated at room temperature 5 min prior to immunization. Mice were challenged (IN) with PR8^H6N1−BR18^ virus at 3 weeks. (B) NA sera antibodies at 3 weeks post-immunization were measured by ELISA (top panel) and displayed as the mean ± SD. NAI antibody titers (bottom panel) were determined by ELLA and are displayed with the mean (bar) ± SD. Both assays were performed with PR8^H1N1/BR18^ virus. *p* values were calculated by assigning 0 to samples below the LOD and using a student unpaired t-test with a 95% CI. (C) Graphs showing the survival (top panel) and mean weight change ±SD (bottom panel) of each group following the PR8^H6N1−BR18^ virus challenge.

There are several manufacturing advantages to modular vaccines as it allows the components to be produced at different sites and makes it easier to exchange a component such as rNA that is likely to change more frequently. Therefore, we examined if the benefits of the cVLP display could be achieved in an admix strategy (Figure 5A). For this test, we shifted the ligation to room temperature (∼22°C) to increase the kinetics (Figure S4) and determined that 5 min resulted in an ∼15% coupling efficiency which corresponds with the display of up to 27 rN1 tetramers per cVLP (Figure S8). Based on these results, we mixed equal volumes of rN1 (0.2 mg/mL) and cVLPs (0.1 mg/mL) and then immunized groups of mice 5 min later. Control groups received 5 μg of rN1 or 2.5 μg of cVLPs. The admix cVLP and rN1 group exhibited higher NA binding and NAI antibody responses than the control groups (Figure 5B). In addition, all the admix-immunized mice survived the lethal viral challenge with some weight loss (Figure 5C), indicating than the rN1 and cVLPs could be provided as a multicomponent vaccine, providing flexibility for seasonal antigen changes.

**Figure 6:**
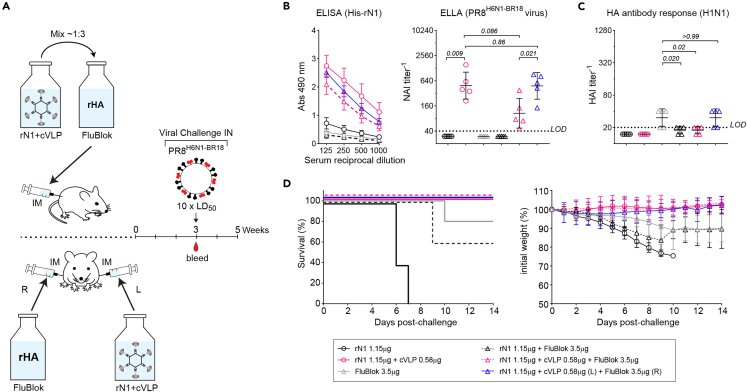
Modularity of rN1 ligated AP205 cVLPs with commercial vaccines (A) Diagram of the admix (top) and left-right co-administration (bottom) strategies for supplementing the commercial influenza vaccine FluBlok with rN1 ligated cVLPs in groups of female DBA/2J mice (*n* = 5). Vials containing rN1 ligated cVLPs (100 μg/mL rN1 content) were mixed 1:3.3 with FluBlok (90 μg/mL content of each rHA) and administered together (top) or mixed with buffer and administered in different (left or right) legs. (B) NA binding sera antibody titers at 3 weeks post-immunization were measured by ELISA (left panel) using His-rN1 protein and are displayed as the mean ± SD. NAI antibody titers (right panel) were determined by ELLA using PR8^H6N1−BR18^ virions and are displayed with the mean (bar) ± SD. ELLA *p* values were calculated by assigning 0 to samples below the LOD and using a student unpaired t-test with a 95% CI. (C) Hemagglutination inhibition (HAI) titers against H1N1 were determined in the indicated groups at 3 weeks post-immunization using the A/Victoria/2570/2019 (H1N1) strain, which is antigenically similar to the H1N1 strain in FluBlok. Titers are displayed with the geometric mean (bar) ± SD. *p* values were calculated by assigning 0 to samples below the LOD and using a student unpaired t-test with a 95% CI. (D) Graphs showing the survival (left panel) and mean weight change ±SD (right panel) of the indicated immunization groups following viral challenge with a 10 × LD_50_ dose of PR8^H6N1−BR18^ virus.

Finally, we tested the immunogenicity of the cVLP-displayed rN1 following admix and co-administration strategies with a commercially available influenza vaccine (Figure 6A). With the rHA vaccine FluBlok, both cVLP-displayed rN1 administration strategies supported increased NA binding and NAI antibody responses (Figure 6B). However, the NAI antibody responses were slightly lower with the admix strategy and a similar pattern was reflected in the HAI responses from FluBlok (Figures 6C and S9). We also subjected these mice to a lethal viral (PR8^H6N1−BR18^) challenge and all the groups that received the cVLP-displayed rN1 with FluBlok survived and showed minimal weight loss, whereas the group that received Flublok alone did not all survive and showed more significant weight loss that did not fully recover (Figure 6D). Additionally, cVLP-displayed rN1 also retained its immunogenicity following admix or co-administration with the adjuvanted egg-based vaccine FLUAD, yielding largely similar results with much higher antibody levels likely due to the presence of adjuvant as well as some NA in the vaccine (Figure S10). Taken together, these results demonstrate that the multivalent viral-like display on cVLPs significantly improves the stability and immunogenicity of soluble rNAs and that the increased immunogenicity is retained during different supplementation strategies with existing influenza vaccines.

## Discussion

Although several studies indicate that NA supplementation is likely to improve influenza vaccine efficacy,^51^^,^^52^^,^^53^ there has been little consideration for how a supplementation approach could be widely implemented. Recent estimates indicate that ∼500 million doses of influenza vaccines are administered annually across the globe,^54^ emphasizing the importance of identifying approaches for producing high quantities of immunogenic NA antigen. Due to the low abundance of NA in viruses, many studies have turned to chimeric rNA antigens like the ones used in this study. However, protection in naive animal models generally requires microgram amounts of these rNAs.^17^^,^^18^^,^^28^^,^^29^ Based on data presented here and elsewhere,^45^ we asked if the immunogenicity of soluble rNAs can be improved by presenting them in a multivalent viral-like display. To create the multivalent display, we used a split-protein bioconjugation system to couple multiple rNA copies onto the surface of AP205 cVLPs that have a diameter of ∼30 nm.^55^ Our results demonstrate that the multivalent rNA display on these cVLPs resulted in higher antibody responses, substantially reducing the protective dose in a mouse viral challenge model. The display also helped to maintain the rNA stability at 4°C for up to 10 months, which is greater than the duration of an influenza season, and the increased antibody response was still observed following different coadministration strategies with commercial vaccines.

Stability and immunogenicity are two of the most critical vaccine qualities and NA is a well-established labile antigen.^7^ Interestingly, the cVLP-display approach improved both the stability and immunogenicity of NA, decreasing the need to identify stabilizing rNA mutations or to add adjuvant. We speculate cVLP coupling stabilizes NA by replicating the assembly and spatial limiting functions of the transmembrane domain,^56^^,^^57^ which is not achieved by the addition of a tetramerization domain alone. In addition, we showed that the coupling minimized the antibody responses to the cVLP scaffold and presumably the tetramerization domain on the soluble rNA, which has previously been shown to be immunogenic.^29^ Importantly, cVLP coupling also improved the immunogenicity of rN2, indicating this display approach is generally applicable to influenza NAs. However, it is not clear if the immunogenicity enhancement by the cVLP display is impacted by the enzymatic functions of NA in the recipient, which can differ between presentations, subtypes and origin.^58^^,^^59^

Our results support the notion that the number or spacing of rNAs per particle can influence the anti-NA antibody response more so than the presence of the cVLPs alone. Specifically, mice immunized with the overnight ligated cVLPs, which contain up to 45 rNA tetramers per cVLP, displayed higher NA antibody titers and less weight loss following viral challenge than the admix ligated cVLPs that contain fewer (up to 27) rNA tetramers per particle. Supporting this conclusion, previous studies with different model antigens have shown that a spacing of 50–100 Å between the antigens on cVLPs is optimal for B cell activation,^60^ as it strengthens the foreign pathogen-associated molecular pattern (PAMP) of the antigen and increases the B cell receptor cross-linking efficiency.^61^ In addition, the increase in the IgG2a antibody response against NA is likely due to the presence of prokaryotic RNA enclosed within the lumen of the cVLP, which has been shown to activate TLR7/TLR8 and promote Th1-type responses in mice.^41^^,^^50^^,^^62^

Silencing immune responses against the cVLP and the ease of incorporating different NAs suggest this approach could be developed into a viable seasonal quadrivalent/trivalent NA vaccine. Supporting this possibility, cVLP coupling can be performed ahead of time or in an admix manner and the rNA immunogenicity was retained following administration with commercial vaccines in admix and co-administration formats, each with its own advantage. While the admix strategy only requires one needle, both the NA and HAI titers were slightly reduced suggesting some interference occurred. In contrast, no interference was seen in the NA and HAI antibody responses with the co-administration strategy, but it involves two needles. However, it is possible that the additional NA response decreases the HA dependence making both approaches viable. Future studies looking at multiple rNA antigens, adjuvant benefits, and the advantages of the combined HA and NA responses, should help to further validate the ability of this approach to potentially enhance the breadth and efficacy of influenza vaccines.

### Limitations of the study

This study primarily investigated the benefits of covalently attaching secreted rNA tetramers from an H1N1 strain to cVLPs. However, current influenza vaccines are recommended to possess HA antigens from two IAV strains (H1N1 and H3N2) and one influenza B strain from the Victoria lineage. While the results showed the cVLP conjugation increased the stability and immunogenicity of rN1 in mice, it is unclear if the same benefits would generally apply to secreted rNA antigens from H1N1, H3N2, and type B strains. It should be noted that we did observe an increase in rN2 immunogenicity, but the stability at 4°C was not monitored due to the lack of established potency reagents. In addition, the methodology used in this study is dependent on producing soluble rNAs with a similar conformation as the native NA and it is conceivable that the antigen design would have to be optimized for each NA, similar to a recent report on rN1.^63^

The observed increase in the rNA immunogenicity is likely due to a combination of the multivalent presentation on the cVLP and the immunostimulatory properties of the prokaryotic RNA associated with the cVLP.^33^^,^^50^ Although we did not specifically differentiate these contributions for rNA, previous work using different antigens have demonstrated that co-formulating unconjugated cVLPs with soluble antigen does not induce similar responses as conjugated cVLPs, underscoring the importance of the multivalent display.^35^^,^^38^^,^^64^^,^^65^ The longevity of the antibody responses and the induction of cell-mediated responses were also not examined in this study but would be an important clinical consideration. Finally, as some interference was observed upon admixing the rNA-cVLPs with commercial vaccines, studies are needed to examine if this is due to non-desirable interactions or excipient incompatibility as minimizing the interference would make this, or any other NA combination vaccine, easier to administer in a single dose.

## STAR★Methods

### Key resources table

**Table:** 

REAGENT or RESOURCE	SOURCE	IDENTIFIER
**Antibodies**		

1H5 mouse monoclonal anti-NA1	Published work^46^	N/A
CD6 mouse monoclonal anti-NA1	Published work^19^^,^^66^	N/A
4C4 mouse monoclonal anti-NA1	Published work^46^	N/A
4E9 mouse monoclonal anti-NA1	Published work^46^^,^^66^	N/A
B10 mouse monoclonal anti-NA2	Published work^67^	N/A
Goat anti-mouse IgG HRP linked secondary antibody	Sigma-Aldrich	Cat# 12–349; RRID: AB_390192
Peroxidase AffiniPure Goat Anti-Mouse IgG, Fcγ subclass 1 specific	Jackson ImmunoResearch	Cat# 115-035-205; RRID: AB_2338513
Peroxidase AffiniPure Goat Anti-Mouse IgG, Fcγ subclass 2a specific	Jackson ImmunoResearch	Cat# 115-035-206; RRID: AB_2338514
Peroxidase AffiniPure Goat Anti-Mouse IgG, Fcγ subclass 2b specific	Jackson ImmunoResearch	Cat# 115-035-207; RRID: AB_2338515
Peroxidase AffiniPure Goat Anti-Mouse IgG, Fcγ subclass 3 specifics	Jackson ImmunoResearch	Cat# 115-035-209; RRID: AB_2338517

**Bacterial and virus strains**		

MAX Efficiency™ DH10Bac Competent Cells	Thermo Fisher Scientific	Cat# 10361012
PR8^H1N1/BR18^	Published work^68^	N/A
PR8^H6N1−BR18^	Published work^45^	N/A
PR8^H6N2−KS17^	Published work^45^	N/A

**Biological samples**		

Fertilized premium eggs	Charles River	Cat# 10100326
FluBlok 2022–2023 Quadrivalent Influenza vaccine	Sanofi	NDC: 49281-723-10; lot#QFAA2232
FLUAD 2022-2023 Quadrivalent Influenza vaccine	Seqirus	NDC: 70461-123-03; lot#346353

**Chemicals, peptides, and recombinant proteins**		

2’-(4-methylumbelliferyl)-α-*d*-*N*-acetylneuraminic acid (MUNANA)	Cayman Chemical	Cat# 16620
Imidazole	Sigma Aldrich	Cat# I2399
Fetuin	Sigma Aldrich	Cat# F3004
*o*-Phenylenediamine dihydrochloride (OPD)	Sigma Aldrich	Cat# P8287
HRP-conjugated peanut agglutinin	Sigma Aldrich	Cat# L7759
Pierce™ Nickel Coated Plate	Thermo Fisher Scientific	Cat# 15442
Immulon® 2 HB 96-Well Microtiter EIA Plate	Immunochemistry Technologies	Cat# 227
Phenyl Sepharose High Performance	CYTIVA	Cat# 17108102
SP Sepharose High Performance	CYTIVA	Cat# 17108702
Lentil Lectin Sepharose 4B	CYTIVA	Cat# 17044401
Novex™ Tris-Glycine Mini Protein Gels, 8–16%, 1.0 mm, WedgeWell™ format	Thermo Fisher Scientific	Cat# XP08160BOX
Novex™ Tris-Glycine Mini Protein Gels, 4–12%, 1.0 mm, WedgeWell™ format	Thermo Fisher Scientific	Cat# XP04122BOX
Catcher-cVLPs	Published work^35^	N/A
Sf-900 III SFM media	Life Technologies	Cat#: 10902088
Agilent AdvanceBio x 300 mm, 2.7 μm, LC column	Agilent	Cat# PL1180-5301

**Critical commercial assays**		

Pierce™ Chromogenic Endotoxin Quant Kit	Thermo Fisher Scientific	Cat# A39552
Quant-iT™ RiboGreen RNA Assay Kit	Thermo Fisher Scientific	Cat# R11490
Quant-iT™ PicoGreen™ dsDNA Assay Kits	Thermo Fisher Scientific	Cat# P11495

**Experimental models: Cell lines**		

Sf9 insect cell	ATCC	CRL-1711

**Experimental models: Organisms/strains**		

DBA/2J #000671 mice, female	Jackson Labs	JAX# 000671

**Recombinant DNA**		

Recombinant NA1 (H1N1 strain A/Brisbane/02/2018) in pFastBac1	This paper	N/A
Recombinant NA2 (H3N2 strain A/Kansas/04/2017) in pFastBac1	This paper	N/A

**Software and algorithms**		

ImageJ	Open source	https://imagej.nih.gov/ij/
GraphPad Prism version 10	GraphPad Software	https://www.graphpad.com

**Other**		

AKTA start	CYTIVA	Cat# 29022094
Bac-to-Bac™ Baculovirus Expression System	Thermo Fisher Scientific	Cat# 10359016
BioTek Cytation 5 Cell Imaging Multimode Reader	Agilent	Cat# Cytation5
Azure Biosystems 600	Azure Biosystems	Cat# AZ1600-01

### Resource availability

#### Lead contact

Further information and requests for resources and reagents should be directed to and will be fulfilled by the lead contact, Robert Daniels (robert.daniels@fda.hhs.gov).

#### Materials availability

This study did not generate unique resources or materials.

#### Data and code availability

• All data reported in this paper will be shared by the lead contact upon request.
• This paper does not report original code.
• Any additional information required to reanalyze the data reported in this paper is available from the lead contact upon request.

### Experimental model and study participant details

#### Ethics statement

All animal experiments were performed with 6 week-old female DBA/2J mice obtained from Jackson labs and were approved by the U.S. FDA Institutional Animal Care and Use Committee (IACUC) under Protocol #2003–18. The animal care and use protocol meets National Institutes of Health (NIH) guidelines.

### Method details

#### Immunization and challenge viruses

HA and NA double-gene reassortant viruses PR8^H1N1/BR18^, PR8^H6N1−BR18^ and PR8^H6N2−KS17^ were all produced by reverse genetics using six internal gene segments from the H1N1 strain A/Puerto Rico/8/1934 (PR8). The HA and NA gene segments in PR8^H1N1/BR18^ are from the H1N1 vaccine strain A/Brisbane/02/2018. HAs in the PR8^H6N1−BR18^ and PR8^H6N2−KS17^ viruses are from the H6N2 strain A/turkey/MA/3740/1965 and the NAs are from the H1N1 strain A/Brisbane/02/2018 (N1-BR18) and H3N2 strain A/Kansas/04/2017, respectively. All viruses were propagated in 10-day old embryonated SPF eggs at 33°C for 3 days prior to harvesting and clarifying (2000 × g; 5 min) the allantoic fluid. Challenge viruses (PR8^H6N1−BR18^ and PR8^H6N2−KS17^) were directly aliquoted and stored at −80°C and the median lethal dose (LD_50_) was determined in 9-week-old female DBA/2J mice.^45^ PR8^H1N1/BR18^ and PR8^H6N1−BR18^ virions for immunization and ELISAs were inactivated with 0.1% beta-propiolactone (BPL) overnight at 4°C and purified as previously described.^68^ Inactivation of purified virions (1 mg/mL) was confirmed by monitoring HA units (HAU) and NA activity in the allantoic fluid of eggs for three consecutive passages using 100 μL (1:10 dilution) inoculums.

#### Recombinant NA (rNA) constructs

rNAs were produced by infecting insect cells with recombinant baculoviruses (BVs) that were created by pFastBac vectors encoding for the soluble rNA. His-rN1 expressing BVs were created by inserting a gene cassette encoding a signal peptide (GP67a), followed by a 6× His-tag, the tetramerization domain (TD) from the human vasodilator stimulating protein, a 7-residue linker and N1 residues 35–469 from the H1N1 strain A/Brisbane/02/2018 with a C49S substitution. rN1 was identical except for the signal peptide (azurocydin) and the exchange of the His-tag with a 15 residue split-protein Tag. The rN2 gene cassette encoded for a signal peptide (azurocydin), followed by the 15-residue Tag, tetrabrachion TD, 7-residue linker and N2 residues 35–469 from the H3N2 strain A/Kansas/14/2017 with a C53S substitution.

#### rNA expression and purification

SF9 insect cells were cultured in Sf-900 III SFM media (Thermo Fisher Scientific) at 27°C and 125 rpm to 2-4 × 10^6^ cells/mL. Cells were infected with BVs expressing rN1 or rN2 at 2% (V/V) for ∼72 h and clarified by sedimentation (4,000 × g; 10 min). Clarified culture medium was adjusted to pH 7.5 with NaOH, passed through a 0.22 μm PES membrane and the rN1 and rN2 were purified by multistep column chromatography as previously described.^48^ Briefly, rN1 was initially captured using Lentil-lectin Sepharose (1 mL/50 mL media). Beads were washed with Buffer A (30 mM Tris pH7.5, 0.5 M NaCl, 1.0 mM CaCl_2_) and proteins were eluted in Buffer A containing 0.5 M α-methyl mannose. Fractions with NA activity were pooled, adjusted to 1.0 M (NH_4_)_2_SO_4_, clarified (100,000 × g; 30 min), applied to a Phenyl-sepharose column and eluted by a 1.0–0 M linear gradient of (NH_4_)_2_SO_4_ in Buffer A. NA active fractions were pooled, exchanged into Buffer B (30 mM MES pH 6.5 and 1.0 mM CaCl_2_), loaded on an SP column and rN1 was eluted with buffer B containing 150 mM NaCl. Fractions with NA activity were pooled and dialyzed against storage buffer (30 mM Tris pH 6.5, 1.0 mM CaCl_2_, 150 mM NaCl, and 2.0% Glycerol). Protein concentration was determined by absorbance (Abs) at 280 nm using the molar extinction coefficient 105850 M^−1^cm^−1^, aliquots were prepared and stored at −80°C. For rN2, the Lentil-lectin eluate was exchanged into Buffer A without NaCl, loaded on a Q column, washed with Buffer A containing 50 mM NaCl and eluted with 200 mM NaCl. Active fractions were pooled, exchanged into Buffer B, loaded on an SP column, washed with Buffer B containing 50 mM NaCl and eluted with 200 mM NaCl. rN2 protein concentration was determined using the molar extinction coefficient 83975 M^−1^cm^−1^ and aliquots were stored at −80°C. His-rN1 protein was expressed in High five insect cells and purified by immobilized metal affinity chromatography using Ni-NTA Sepharose beads and imidazole for elution.^28^

#### Expression and purification of AP205 Catcher-cVLPs

Catcher-cVLPs were designed by fusing a proprietary Tag/Catcher split-protein pair to the N-terminus of the Acinetobacter phage AP205 coat protein subunit (Gene ID: 956335).^35^ Catcher-cVLPs were expressed in *E. coli* One Shot BL21 (DE3) cells (Invitrogen) and the resulting self-assembled particles were purified from *E.coli* cell lysate by ultracentrifugation using a Optiprep (Sigma-Aldrich, St Louis, MO, USA) density step gradient. Fractions containing AP205 cVLPs were dialyzed overnight against 5 L PBS pH 7.4 using a 1,000 kDa MWCO membrane (Spectrum Labs). Samples were treated with Triton X-114 and phase separated to reduce LPS.^69^ Protein concentration was determined by BCA assay according to the manufacturer’s instructions (Thermo Scientific), and the samples were aliquoted and stored at −80°C.

#### Neuraminidase activity assay

Enzymatic activity was measured using 2’-(4-methylumbelliferyl)-α-*d*-N-acetylneuraminic acid (MUNANA). rNAs were diluted to ∼5 μg/mL and 10 μL were added to 96-well, black wall, clear bottom plates (Corning). Reactions were initiated by adding 190 μL of 37°C substrate solution (5 μL of 2 mM MUNANA and 185 μL 0.1 M KH_2_PO_4_ pH 6 containing 1 mM CaCl_2_) per well. Reactions were monitored at 30 s intervals on a Cytation 5 Plate Reader (Biotek) at 37°C for 10 min using 365 nm (excitation) and 450 nm (emission) wavelengths. NA activity was determined by the initial linear velocity in the emission versus time graph and reported either as RFU/sec or pmol/sec, which was determined using a 5-Methylumbelliferone standard.

#### Endotoxin measurements

Pierce Chromogenic Endotoxin Quant Kit was used for endotoxin measurements according to the manufacturer’s instruction. Briefly, rN1 (80 μg/mL) and cVLPs (560 μg/mL) were serially diluted 1:5 and added (50 μL/well) along with standards to a 96-well plate that was preheated at 37°C. Amebocyte Lysate Reagent (50 μL/well) was added and the plate was incubated at 37°C for ∼15 min. Chromogenic substrate (100 μL/well) was added and samples were incubated 6 min at 37°C. Reactions were stopped with 25% (v/v) acetic acid (50 μL/well) and the absorbance at 405 nm was measured. Endotoxin concentrations were determined using the standard curve and the amounts in each dose were calculated.

#### RNA and DNA measurements

rN1 (0.421 mg/mL) and cVLP (5.6 mg/mL) preparations were serially diluted 1:5 and the RNa and DNA measurements were performed using the Invitrogen Quant-it RiboGreen RNA and Quant-iT PicoGreen dsDNA Assay Kits (Thermo Fisher Scientific, Waltham, MA). The high-range assay (1 ug/mL to 10 ng/mL) was used for both assays along with a standard following the manufacturer’s protocols. Fluorescence was measured with a Cytation 5 Plate Reader using 480 nm (excitation) and 520 nm (emission) wavelengths. RNA and DNA concentrations in the samples were determined from the standard curve and the amounts in each dose were calculated.

#### cVLP conjugations and analysis by SDS-PAGE

Conjugation optimizations were carried out in 50 μL volumes by combining a fixed amount of cVLP (2 μg) with the indicated amount of rN1 or rN2 in PBS pH 7.2. Incubations were performed at the indicated temperatures and times. Immunization antigen preparations were carried out in 5 mL volumes that either contained 0.1 mg/mL of rNA (control), 0.05 mg/mL VLP (control), or 0.05 mg/mL cVLP and 0.1 mg/mL of rNA. All preparations were made in sterile PBS pH 7.2 and the incubations were performed at 4°C for ∼18 h, prior to aliquoting and storing at both 4°C and −80°C. Optimization of mix-and-shoot (admix) room temperature conjugations were carried out by mixing 25 μL of PBS pH 7.2 containing 2 μg of cVLP with 25 μL of PBS pH 7.2 containing 4 μg of rN1 and incubating for the indicated time prior to adding boiling SDS-PAGE sample buffer. cVLP coupling was monitored by reducing SDS-PAGE using 8–16% Tris-Glycine Novex WedgeWell gels (Thermo Fisher Scientific). Protein bands were visualized by staining with SimplyBlue (Thermo Fisher Scientific), gels were captured using a C600 Azure biosystems imager and band density calculations were performed using ImageJ. Inhibition of the AP205 catchers was performed by adding a 15-residue peptide corresponding to the Tag sequence at a molar ratio of 20:1 with the cVLPs and incubating the mixture for 4 h at room temperature prior to the overnight 4C rN1 incubation.

#### Transmission electron microscopy (TEM)

Unligated cVLP (0.56 mg/mL) and rN1 + cVLP (1:1 ratio, total protein 0.04 mg/mL) samples were analyzed by TEM to verify particle integrity. Carbon grids (200-mesh) were glow discharged for 30 s at 15 mA. Undiluted samples (5 μL) were adsorbed to the grids and incubated for 1 min. Excess sample was removed from the grid with filter paper prior to staining with 2% uranyl acetate for 45 s. Excess stain was similarly removed with filter paper and the stained grids were analyzed using a Morgagni 268 transmission electron microscope. Images were recorded for 1000 ms at 70× magnification or higher.

#### Dynamic light scattering (DLS)

Undiluted samples (70 μL) of Tag-rN1 + cVLP (1:1 ratio, total protein 0.04 mg/mL) and unligated cVLP (0.56 mg/mL) were added to a disposable cuvette and analyzed using a Wyatt technologies DynaPro Nanostar instrument (658 nm laser). Measurements were performed using 20 acquisitions of 5 s at 25°C. Each measurement was repeated twice. Percent intensity size distributions were obtained from a regularization fit using Wyatt DYNAMICS 7.10.0.21 software. Average particle diameters and polydispersity (%Pd) of triplicates were recorded for the main peak.

#### Size exclusion chromatography (SEC)

SEC was performed with an AdvanceBio SEC 300 Å (7.8 × 300 mm, 2.7 μm) column (Agilent) using an Agilent 1260 prime high-performance liquid chromatography system equipped with variable-wavelength detector, and fraction collector. Samples (15 μL) were injected and run using a flow rate of 1 mL/min and a mobile phase consisting of 30 mM Tris, pH 6.5, 150 mM NaCl, and 1 mM CaCl_2_. Fractions (200 μL) were collected for rNA activity analysis.

#### Intramuscular (IM) immunizations

Groups of six-week-old DBA/2J female mice (*N* = 5) were immunized with 50 μL doses containing either 1 μg of BPL inactivated PR8^H1N1/BR18^ virions or His-rN1 protein that were prepared in in sterile PBS pH 7.2 containing 1 mM CaCl_2_. For rN1, rN2, cVLP, rN1+cVLP or rN2 + cVLP immunizations, aliquots stored at −80°C which contained 0.1 mg/mL of rNA and 0.05 mg/mL of cVLP where indicated were thawed and administered directly for the high dose (5 μg rNA). The two lower doses (1.25 μg and 0.31 μg rNA) were prepared by two 4-fold serial dilutions of the proteins in sterile PBS pH 7.2 prior to administration. All doses were administered in 50 μL volumes by IM injection with a 28.5-gauge needle (Exelint International Co.). Control groups (*N* = 5) were injected with sterile PBS pH7.2 or the indicated amount of BSA (50 μL/mouse). Tail bleed samples were collected 3-week post-immunization for serological analysis. Immunizations with rN1 and rN1 + cVLP for IgG subclass analysis were performed with 4 mice per group. Admix immunizations with rN1 and cVLP were carried out by mixing equal volumes of 0.2 mg/mL rNA with 0.1 mg/mL of cVLP or sterile PBS pH7.2 (control) and incubating at room temperature for 5 min prior to administration by IM injection into groups of six-week-old DBA/2J female mice (*N* = 4). For admix immunizations with rN1 + cVLP and commercial vaccine, rN1 (0.1 mg/mL) and rN1 + cVLP (0.1 mg/mL + 0.05 mg/mL) aliquots stored at −80°C were thawed and 1 volume was added to 3.3 volumes of commercial vaccine (0.09 mg/mL per HA for FluBlok and 0.03 mg/mL per HA for FLUAD) or 3.3 volumes of sterile PBS pH 7.2 (controls). One volume of sterile PBS pH 7.2 was added to 3.3 volumes of commercial vaccine for an additional control. All doses were then administered in 50 μL volumes by IM into groups of six-week-old DBA/2J female mice (*N* = 4). For left (L) and right (R) administrations, 1 volume of the rN1 + cVLP (0.1 mg/mL + 0.05 mg/mL) aliquot was added to 3.3 volumes of sterile PBS pH 7.2 and 1 volume of sterile PBS pH 7.2 was added to 3.3 volumes of commercial vaccine prior to administering 50 μL of each by IM injection into the left and right hind legs, respectively.

#### Viral challenge

Mice were intranasally (IN) infected with 10 × LD_50_ of recombinant PR8^H6N1−BR18^ virus or PR8^H6N2−KS17^ virus diluted in 50 μL of sterile PBS pH 7.2. Weight change and survival were recorded over two-week period and moribund mice or mice that showed 25% weight loss were euthanized by CO_2_ in accordance with the institutional Animal Study Proposal at 30% per minute.

#### Enzyme-linked immunosorbent assays (ELISAs)

Antigenicity and stability indicating ELISAs were performed using Immunol 2HB flat bottom 96-well microtiter plates (Thermo Fisher Scientific).^70^ Wells were coated overnight at 4°C with N1 specific capture mAbs CD6, 4C4, or 1H5 (0.5 μg/well) in KPL coating solution (SeraCare). Plates were washed 3 times with PBST (1× PBS pH 7.4 containing 0.05% Tween 20) and blocked 1 h at 37°C with PBST containing 15% fetal bovine serum (Sigma). Blocking solution was discarded, rNA samples (initial amount 0.1 μg/well followed by 1:2 serial dilutions) were added to the wells incubated 1 h at 37°C. Wells were washed and incubated with HRP-conjugated N1 detection mAbs 4E9 (CD6 or 4C4 capture) or 4C4 (1H5 capture) at 37°C for 1 h. Plates were washed and developed using *o*-phenylenediamine dihydrochloride (OPD; Sigma) for 10 min. Reactions were stopped by the addition of 1 N H_2_SO_4_ and Abs at 490 nm were read immediately. ELISAs for measuring N1 antibody responses with PR8^H6N1−BR18^ or PR8^H1N1/BR18^ viruses were performed on Immunol 2HB plates coated with the indicated purified virus at a concentration of 1.0 μg/well total viral protein. ELISAs using His-rN1 were performed on Nickel coated plates (Pierce) that were incubated for 2 h at room temperature with His-rN1 (300 ng/well) which contains the head domain residues (amino acids 82–469) of the NA from the H1N1 strain A/Brisbane/02/2018. Plates were washed, 2-fold serially diluted serum samples (125-fold initial dilution) were added, and the plates were incubated for 1 h at 37°C. Plates were washed, incubated with an HRP-labeled secondary against mouse IgG (1:10,000 dilution per well) for 1 h at 37°C and developed using OPD as described above. ELISAs for examining IgG subclasses were performed on 2HB plates coated with purified PR8^H1N1/BR18^ virus (1.0 μg/well) in KPL solution overnight at 37°C. Plate processing, serum sample addition and detection are described above except that HRP-labeled secondaries against mouse IgG1, IgG2a, IgG2b and IgG3 (Jackson ImmunoResearch Laboratories) were used for detection. N2 antibody responses were measured similarly using 2HB plates coated with rN2 (100 ng/well). Endpoint titers were determined by the lowest dilution with an Abs 490 nm value ≥0.1. Sera with all values below the LOD were treated as 0 for statistical analysis.

#### NA inhibitory (NAI) antibody titer determination

NAI antibody titers were measured by a fetuin-based (2.5 μg/well) enzyme-linked lectin assay (ELLA).^49^ Briefly, sera were heat treated 45 min at 56°C and 2-fold serial dilutions starting at 1:20 or 1:40 were made using 2-(N-morpholino)ethanesulfonic acid (MES) buffer (30 mM MES pH 6.5, 150 mM NaCl and 20 mM CaCl_2_) containing 1% BSA (Sigma), and 0.5% Tween 20. Diluted sera samples were mixed with a predetermined amount of PR8^H6N1−BR18^, PR8^H1N1/BR18^ or H1N1^CA09^ virus as indicated which yields an Abs 490 nm ∼ 1–2.0 in the assay. Mixed samples were added to the 96-well fetuin coated Maxisorp plates, incubated ∼18 h at 37°C, washed with PBST, and incubated with HRP-conjugated peanut agglutinin (Sigma) for 2 h in the dark at room temperature. Plates were washed with PBST, developed using OPD and read as described for the ELISAs. ELLA performed with His-rN1 that contains the head domain residues (amino acids 82–469) of the NA from the H1N1 strain A/Brisbane/02/2018 were carried out similarly except the incubation period on the fetuin coated plates was extended to 48 h to minimize the NA amount in the assay. All data were plotted in GraphPad Prism 8.0 software. NAI titers or the reciprocal of the serum dilution that yields ∼50% inhibition of NA activity was determined using a four-parameter non-linear regression analysis with bottom and top constraints that equal the average of the background and virus only control wells.

#### Hemagglutinin inhibition assay (HAI)

Serum samples (10 μL) were treated with 30 μL of Receptor Destroying Enzyme (RDE) Kit (Cosmos Biomedical Ltd) overnight at 37°C and heat inactivated at 56°C for 45 min. Treated sera were brought up to 100 μL using PBS pH 7.2. 2-fold serial dilutions of the treated sera were prepared in each row of a 96-well U-bottom plate containing 25 μL of PBS pH 7.2 in each well. The indicated viruses (A/Victoria/2570/2019 (H1N1; IVR-215), A/Darwin/9/2021 (H3N2; IVR-228), B/Austria/1359417/2021 (B Victoria lineage; BVR-26)) were diluted in PBS pH 7.2 to 4 HAU, which was predetermined, and 25 μL were added to each well. Plates were incubated 30 min at room temperature before adding 50 μL of 0.5% TRBCs in PBS pH 7.2 to each well and incubating for an additional 45 min. HAI titers were calculated based on the lowest reciprocal sera dilution that prevented hemagglutination.

### Quantification and statistical analysis

Data analysis was performed using GraphPad Prism 10 software. All *p* values were either determined using a parametric Student unpaired t-test with a two-tailed analysis and a confidence interval (CI) of 95% or a two-way ANOVA using a Tukey multiple comparisons test with single pooled variance and a 95% CI.
