# Supplementary material for: Capsid virus-like particle display improves recombinant influenza neuraminidase antigen stability and immunogenicity in mice
Source: iScience. 2024 May 20;27(6):110038. doi: 10.1016/j.isci.2024.110038 (PMC11179578; doi:10.1016/j.isci.2024.110038)
Supplement: Document S1. Figures S1–S10 [file mmc1.pdf]

iScience, Volume *27*

Supplemental information

Capsid virus-like particle display improves recombinant influenza neuraminidase antigen stability and immunogenicity in mice

Hyeog Kang, Mira Rakic Martinez, Kara-Lee Aves, Anna Kathrine Okholm, Hongquan Wan, Sylvie Chabot, Tahir Malik, Adam F. Sander, and Robert Daniels

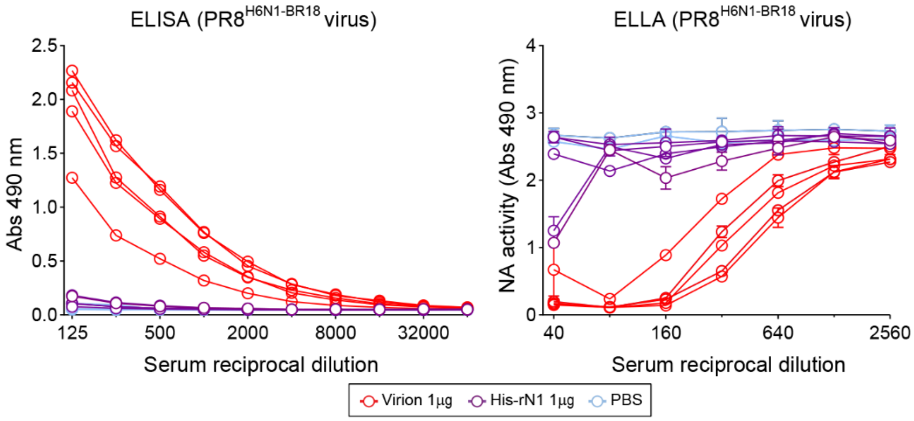

Supplemental Figure 1. NA antibody responses in mice immunized with inactivated virions or rNA, Related to Figure 1C. DBA/2J mice were immunized with 1 μg of BPL inactivated PR8$^{H1N1/BR18}$ virions or His-rN1 protein. Sera collected 3 weeks post-immunization were assessed for NA antibodies by ELISA (left panel) and NAI antibodies by ELLA (right panel). Both assays were performed with recombinant PR8$^{H6N1-BR18}$ virus. ELLA analysis was performed in duplicate, and the mean values are displayed ± SD.

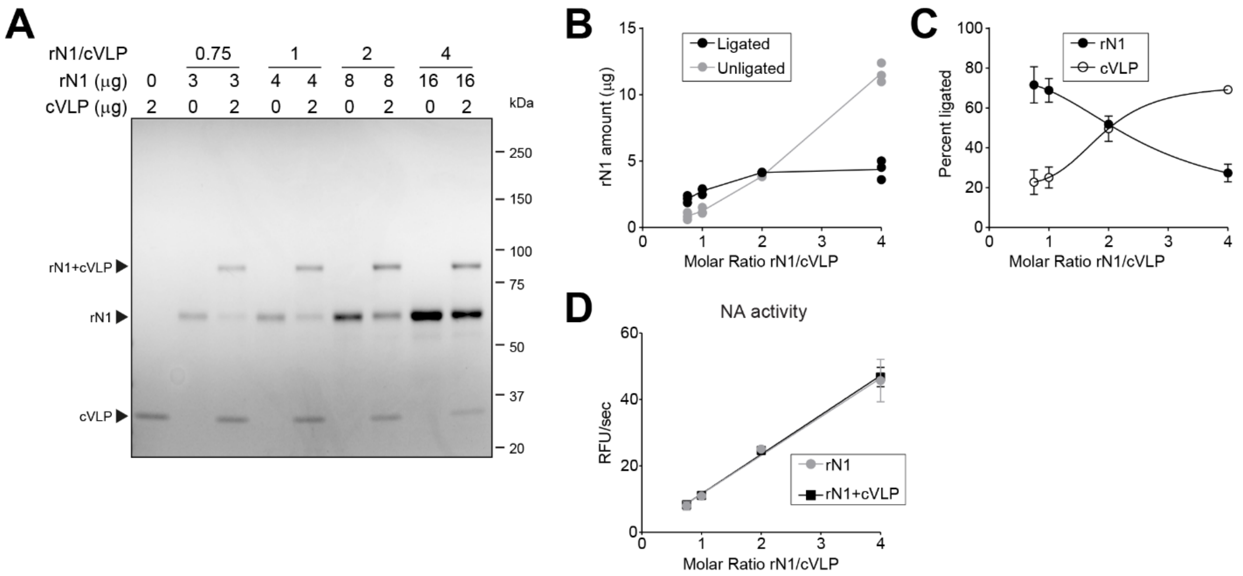

Supplemental Figure 2. rN1 and cVLP ligation kinetics at different molar ratios, Related to Figure 2.

A. Coomassie stained reducing SDS-PAGE gel showing equal volumes cVLP ligations that were performed at of 4ºC for ~18 h using fixed cVLP amounts and increasing rN1 amounts. Bands corresponding to the cVLP, rN1 and cVLP ligated rN1 are indicated. **B**. Graph showing the calculated amounts of unligated rN1 and cVLP ligated rN1 from each condition. Unligated rN1 amounts were determined by multiplying the total rN1 amount in each sample by the corresponding rN1 band density normalized to the no cVLP control band density. Ligated amounts were calculated by subtraction this value from the total rN1 amount in each sample. Data are from three technical repeats. **C**. Graph displaying the percentage of ligated cVLP and rN1 at each ligation condition. Mean values from three technical repeats are plotted ± SD. **D**. NA activities are shown for each of the rN1 mock and cVLP ligation samples. Measurements were performed using equal sample volumes (1.5 μl). Mean values from three technical repeats are plotted ± SD.

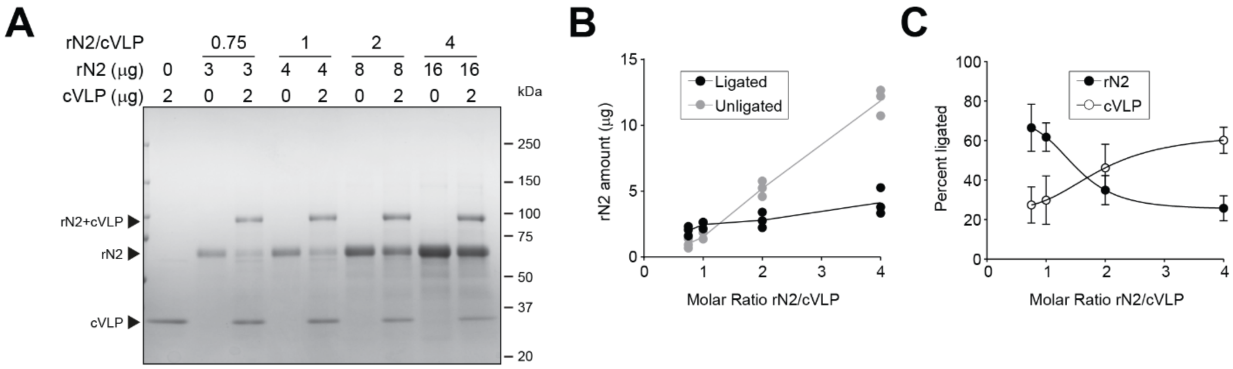

Supplemental Figure 3. rN2 and cVLP ligation kinetics at different molar ratios, Related to Figure 2.

A. Coomassie stained reducing SDS-PAGE gel showing equal volumes of ~18 h cVLP ligations that were performed at 4ºC with fixed cVLP amounts and increasing rN2 amounts. Bands corresponding to the cVLP, rN2 and cVLP ligated rN2 are indicated. **B.** Unligated and cVLP ligated rN2 amounts from each condition were calculated as described in Supplemental Figure 2B. Data are from three technical repeats. **C**. Graph displaying the percentage of ligated cVLP and rN2 in each ligation condition. Data are means ± SD from two technical repeats.

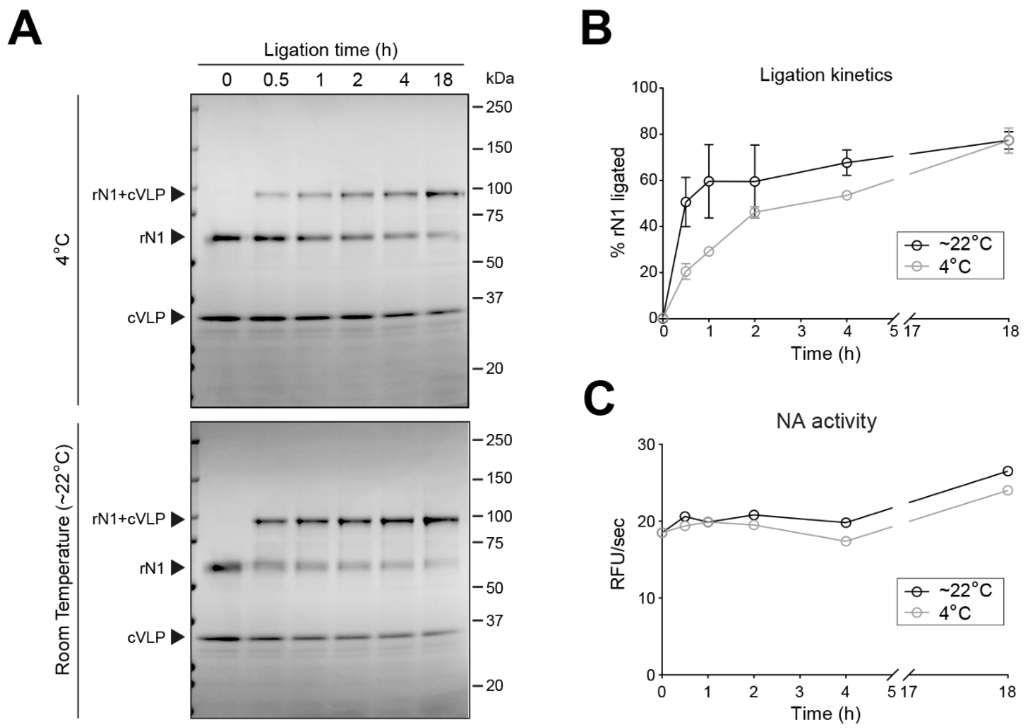

Supplemental Figure 4. Temperature influence on rN1 and cVLP ligation kinetics, Related to Figures 2 and 5. **A.** rN1 was mixed with cVLPs at a molar ratio of 1:1 and incubated at either 4ºC (top gel) or room temperature (lower gel). Samples at the indicated ligation times were inactivated by adding boiling sample buffer, resolved by reducing SDS-PAGE gel and visualized by Coomassie staining. Bands corresponding to the cVLP, rN1 and cVLP ligated rN1 are indicated. **B**. Percentage of ligated rN1 in each sample was calculated by normalizing the band density of the unligated rN1 band in each sample by the 0 h control band, subtracting the value from 1 and multiplying by 100%. Data are means ± SD from two technical repeats. **C**. NA activity in each sample was measured directly at each time point using equal sample volumes (1.5 µl) and MUNANA. Data are from a single experiment.

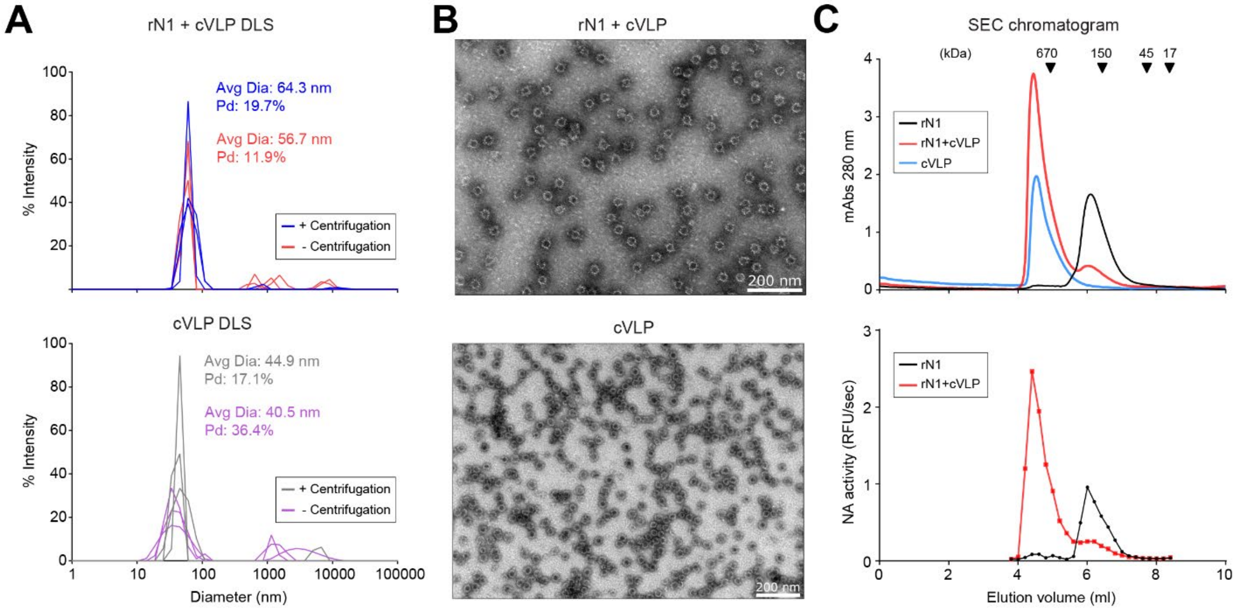

Supplemental Figure 5. Size and integrity of rN1 ligated cVLPs, Related to Figure 2.

A. Size distributions of rN1 ligated cVLPs stored at -80ºC were measured by dynamic light scattering (DLS) and compared to the unligated cVLPs before and after centrifugation. Three independent measurements are displayed for each sample with the average diameter and polydispersity (Pd) of the major peak. Data from the centrifuged samples are shown in Figure 2G of the manuscript. **B**. Representative TEM images of rN1 ligated cVLPs and unligated cVLPs stored at -80ºC. Subsections of each image are displayed in Figure 2H of the manuscript. **C**. rN1, cVLP and rN1 + cVLP samples stored at -80ºC were separated by size exclusion chromatography (SEC) and collected in 200 µl fractions. Protein elution profiles (upper graph) are displayed with the NA activity in the fractions (lower graph). Arrows indicate elution peaks of the molecular weight standards.

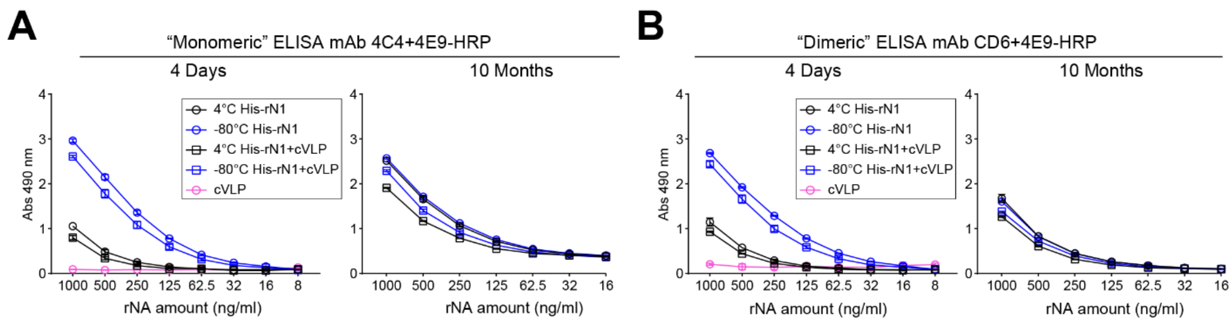

Supplemental Figure 6. Soluble His-rN1 shows significant antigenic changes during 4ºC storage, Related to Figure 3. "Monomeric" (**A**) and "Dimeric" (**B**) N1 capture sandwich ELISAs were used to monitor antigenic changes in His-rN1 following storage at 4ºC or -80ºC for 4 days and 10 months. Data is the mean ± SD from two technical repeats.

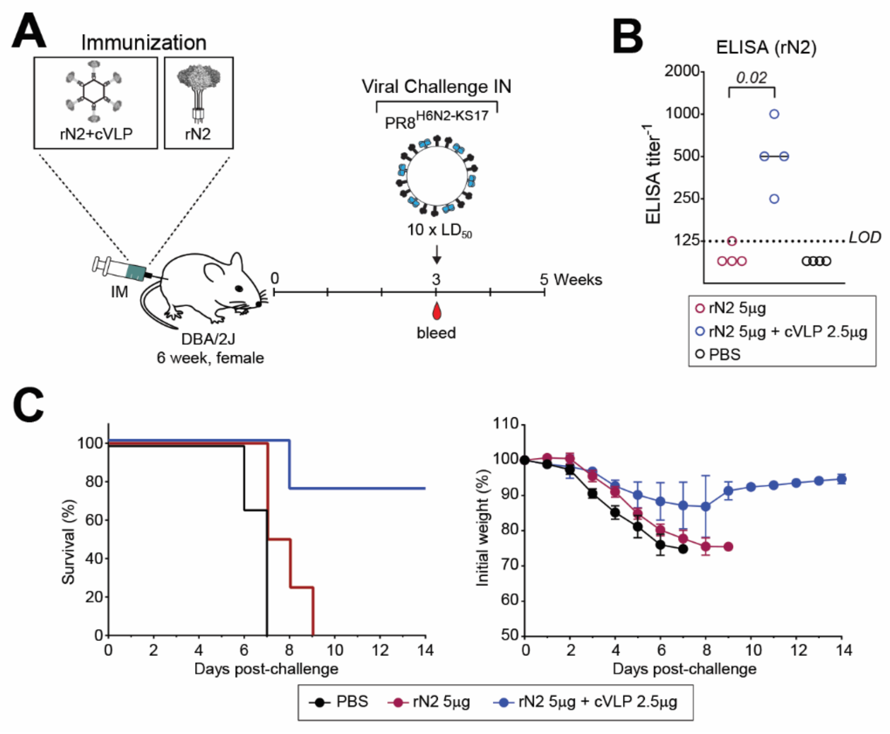

Supplemental Figure 7. **Immune responses to rN2 and cVLP ligated rN2 in mice, Related to Figure 4.**

A. Immunization and lethal virus challenge scheme for comparing antibody responses to unligated and cVLP ligated rN2 in DBA/2J mice. **B**. N2 sera antibody titers 3-weeks post-immunization were measured by an endpoint ELISA using rN2 protein. The assay was performed in duplicate and the average titer for each sera is displayed with the mean (bar) for each group. *P* values were calculated by a student unpaired t-test with a 95% CI. Samples below the limit of detection were assigned 0 for the anlaysis. **C**. Graphs displaying the survival (left panel) and mean weight change (right panel) in each group following the lethal PR8$^{H6N2\text{-}KS17}$ viral challenge.

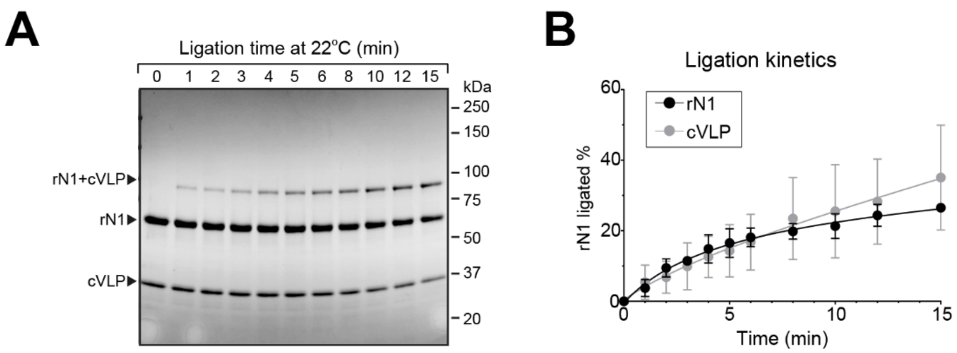

Supplemental Figure 8. rN1 ligation kinetics to AP205 cVLPs at room temperature, Related to Figure 5.

A. Coomassie-stained reducing SDS-PAGE gel displaying the rN1 temporal ligation kinetics with cVLPs at room temperature. Bands corresponding to the cVLP, rN1 and cVLP ligated rN1 are indicated. **B**. Graph displaying the percentage of ligated rN1 and cVLP at the indicated room temperature incubation times. Data are means ± SD from two independent biological replicates.

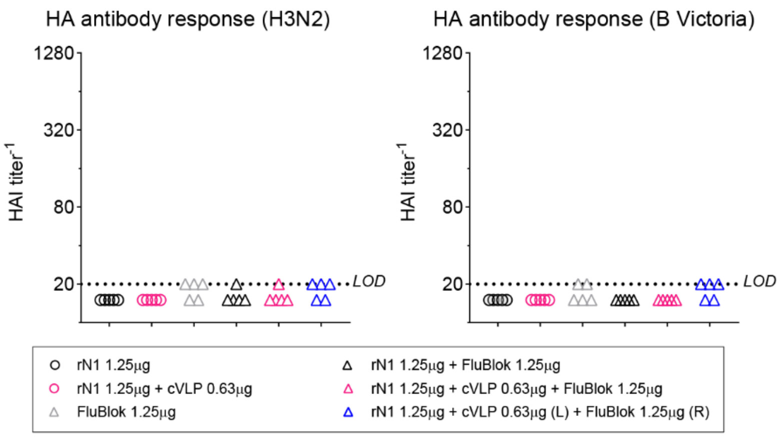

Supplemental Figure 9. FluBlok HA antibody responses following supplementation with rN1 displayed on cVLPs, Related to Figure 6. HAI sera antibody titers against H3N2 (left panel) and the B Victoria lineage (right panel) were measured 3 weeks post-immunization using A/Darwin/9/2021 (H3N2) and B/Austria/1359417/2021 viruses, respectively. Both viruses are antigenically similar to the HA antigens present in the FluBlok used in the experiment.

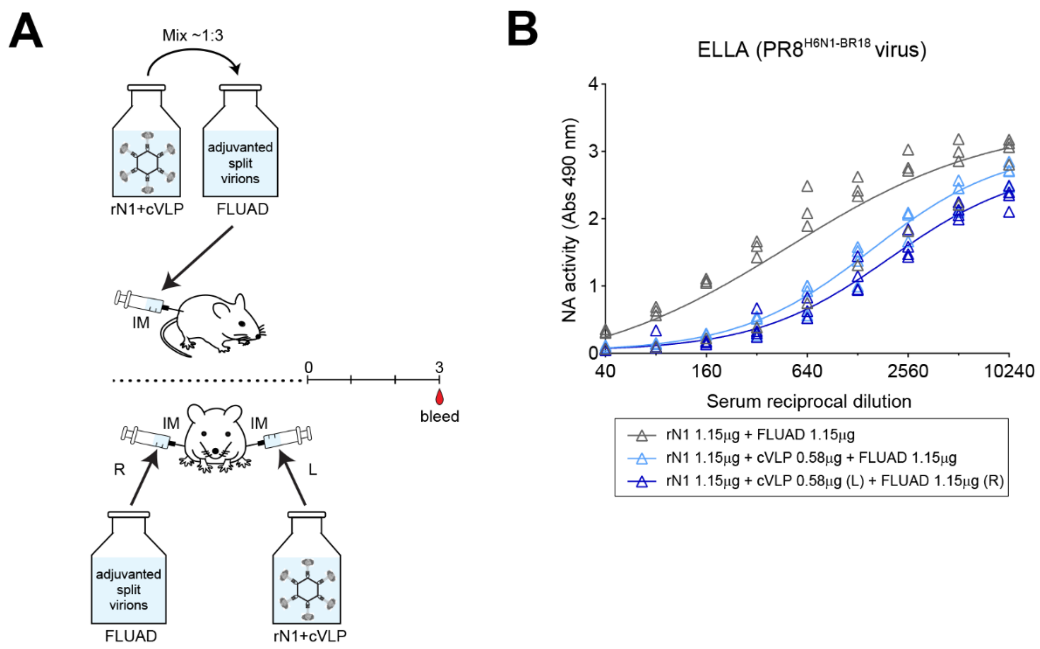

Supplemental Figure 10. Immunogenicity of cVLP displayed rN1 after co-administration with FLUAD, Related to Figure 6. A. Diagram of the admix (top) and left-right co-administration (bottom) strategies for supplementing the commercial egg-based adjuvanted influenza vaccine FLUAD with rN1 ligated cVLPs. Vials containing rN1 ligated cVLPs (100 µg/ml rN1 content) were mixed 1:3.3 with FLUAD (30 µg/ml content of each HA) and administered together (top) or mixed with buffer and administered in different legs (bottom). **B**. NAI sera antibody responses at 3 weeks post-immunization with the different schemes were measured by ELLA using PR8$^{H6N1\text{-}BR18}$ virions. Individual results from each sera dilution are displayed with the Variable slope four parameter nonlinear fit curve (line).
